# Nasal displacement of retinal vessels on the optic disc in glaucoma associated with a nasally angled passage through lamina cribrosa

**DOI:** 10.1038/s41598-021-83720-0

**Published:** 2021-02-18

**Authors:** Yu Sawada, Makoto Araie, Hitomi Shibata, Takeshi Iwase

**Affiliations:** 1 Department of Ophthalmology, Akita University Graduate School of Medicine, 1-1-1 Hondo, Akita, 010-8543 Japan; 2 Kanto Central Hospital of the Mutual Aid Association of Public School Teachers, Tokyo, Japan

**Keywords:** Diseases, Medical research

## Abstract

To investigate nasal displacement of central retinal vessel (CRV) on the optic nerve head (ONH) in glaucoma in association with its passage through lamina cribrosa (LC). This cross-sectional study included 113 eyes with glaucoma and 60 normal eyes. Horizontal spectral-domain optical coherence tomography (SD-OCT) scans of the ONH were acquired, and point where CRV emerged on the ONH surface was defined as the position of the CRV. Next, radial scans of the ONH were acquired, and angle of the CRV passing through the LC was measured. These parameters were compared between glaucomatous and normal eyes by *t*-test, and their relationship with possible confounders was assessed by multiple regression analyses. In glaucoma, CRV was significantly more nasally displaced than it was in normal eyes (66.0 ± 8.6 vs. 54.3 ± 9.5, *P* < 0.0001), and eyes with more vessel displacement exhibited significantly worse glaucomatous visual field defects (*P* = 0.0004). Greater nasal displacement of the CRV was significantly associated with a more nasally angled path through the LC (*rs* = 0.569, *P* < 0.0001). By using SD-OCT, we confirmed that nasal displacement of the CRV on the ONH was associated with glaucoma and was induced by its nasally angled path through the LC.

## Introduction

Eyes with glaucoma present characteristic deformation of the optic nerve head (ONH), including focal or general thinning of the rim tissue and consequent enlargement of the cup^1–3^. Glaucoma is diagnosed based on these ONH deformations and corresponding visual field (VF) defects. Nasal displacement of the central retinal vessel (CRV) on the ONH is known as one of these glaucomatous features. However, very few studies have investigated the position of the CRV on the ONH in glaucoma. The last study conducted on this topic was in 1969 by using fundus photographs^4^, and as far as we are aware, there has been no further study over the ensuring 50 years. Therefore, there is not enough evidence to determine whether the CRV actually displaces nasally in glaucoma, and whether this has any diagnostic implication.

The position of the CRV on the ONH identified in the fundus photograph is the point where it leaves the lamina cribrosa (LC) and emerges on the surface of the ONH. The CRV takes a path through the LC before it emerges on the ONH surface^5^. Therefore, we hypothesized that if the CRV was displaced nasally on the ONH, this was supposedly caused by its nasally shifted path during its passage through the LC. The path of the CRV through the LC has not been studied previously, probably because of the difficulty in observing the LC with the previous examination devices. Recently, spectral-domain optical coherence tomography (SD-OCT) and enhanced-depth imaging (EDI) techniques were invented^6–8^, and it became possible to observe the CRV within the LC in living human eyes.

Therefore, the present study aimed to investigate the position of the CRV on the ONH in glaucoma in association with its passage through the LC, by using SD-OCT.

## Methods

This study was approved by the Institutional Review Board of Akita University Graduate School of Medicine. It was performed with the written informed consent of the participants, and followed the tenets of the Declaration of Helsinki.

The consecutive patients who visited the glaucoma clinic of Akita University Graduate School of Medicine between May 2018 and February 2020 were recruited. Each participant underwent a comprehensive ophthalmic assessment, including refraction tests, measurement of best-corrected visual acuity, central corneal thickness (CCT) and axial length measurement (SP-3000; Tomey Corporation, Nagoya, Japan), Goldmann applanation tonometry, slit lamp biomicroscopy, gonioscopy, stereoscopic examination of the optic discs, color fundus stereo photography (Canon, Tokyo, Japan), Humphrey visual field (VF) test by using the 24–2 Swedish Interactive Threshold Algorithm standard program (Carl Zeiss Meditec, Dublin, CA, USA), and SD-OCT (Spectralis, Heidelberg Engineering GmbH, Heidelberg, Germany). In the pseudophakic eyes, refraction error before surgery was employed. The SD-OCT images were acquired within 3 months of the VF test.

Eyes with open-angle glaucoma (OAG) were included in the study. They were diagnosed as having OAG if they presented an open iridocorneal angle, glaucomatous ONH alternations such as localized or diffuse rim thinning and retinal nerve fiber layer defects, and glaucomatous VF defects corresponding to the glaucomatous structural alternations. Glaucomatous VF defects were defined based on glaucoma hemifield test results outside the normal range, or the presence of at least three contiguous test points within the same hemifield on the pattern deviation plot at *P* < 5%, with at least one of these points at *P* < 1%, which was confirmed by two consecutive reliable tests (fixation loss rate, ≤ 20%; false-positive and false-negative error rates, ≤ 15%). The exclusion criteria were as follows: (1) eyes with poor-quality OCT images in which the path of the CRV through the LC was not clearly presented; (2) eyes with moderate to high myopia (spherical equivalent < -4.0 diopter and axial length > 25.0 mm) to eliminate the effect of myopic deformation of the LC^9–11^; (3) retinal or neuro-ophthalmologic disease that might affect the VF; and (4) congenital optic disc abnormalities and suspected anomalies^12^. Normal eyes were included as controls if they exhibited an intraocular pressure (IOP) ≤ 21 mmHg, an open iridocorneal angle, a normal-appearing optic disc, and no VF defects.

### Observation of the path of the central retinal vessel through the lamina cribrosa

The SD-OCT images of the ONH were acquired using the EDI technique, which improves visualization of the deeper structures by increasing the signal strength and image contrast^6–8^. The magnification error was corrected by using the formula on the basis of the autorefraction keratometry results and focus setting during image acquisition. The scaling of the OCT images was corrected to 1:1 μm before evaluation.

**Figure 1:**
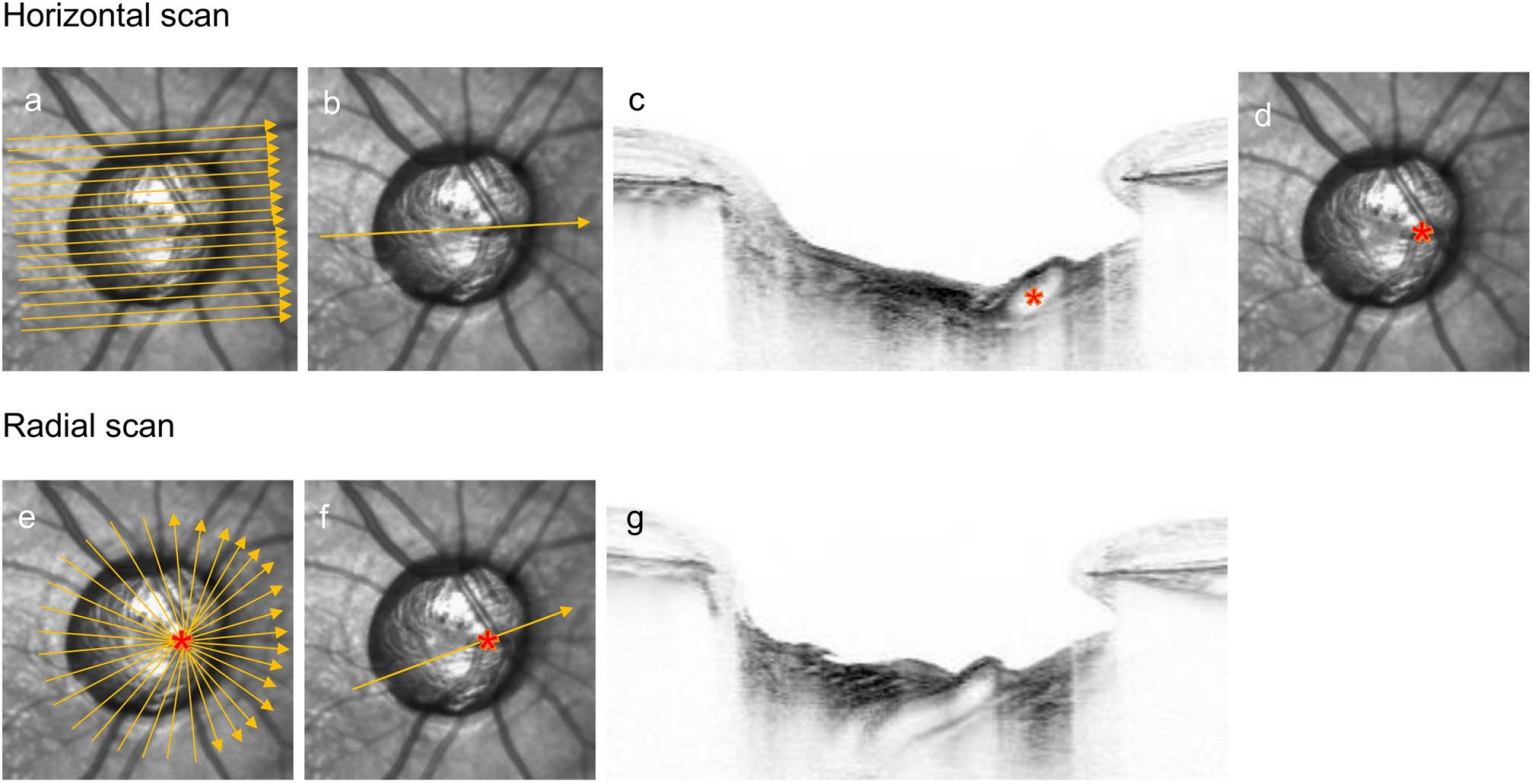
Captured path of the central retinal vessel (CRV) through the lamina cribrosa (LC). (**a**) Horizontal scans of the optic nerve head (ONH) were obtained by alignment to the line that connected the fovea and the disc center (orange arrows). (**b**) Among the horizontal scans, a line that captured the point at which the CRV left the LC to the prelaminar ONH region was identified (orange arrow). (**c**) B-scan image obtained at the scan line shown in (**b**). The red star indicates the point at which the CRV left the LC and emerged on the prelaminar ONH region. (**d**) The point identified in (**c**) was plotted on the corresponding location on the infrared fundus image (red star). (**e**) Radial scans of the ONH were obtained centered on the point at which the CRV left the LC identified in the horizontal scans (red star). (**f**) Among the radial scans, a line that captured the path of the CRV was identified (orange arrow). (**g**) B-scan image that captured the path of the CRV through the LC obtained at the scan line shown in (**f**).

The path of the CRV within the LC was assessed by using SD-OCT B-scan images (Fig. 1). First, horizontal scans of the ONH were acquired by alignment to the line that connected the fovea and the disc center. The horizontal scan lines were 30 μm apart, and each B-scan image was constructed using 35 frames^13–15^. Based on these horizontal scans, the point at which the CRV left the LC to the prelaminar ONH region was identified. Then, radial scans of the ONH were acquired centered on this point, including 48 B-scan images, 3.75 degrees apart, with each B-scan image constructed using 42 frames. Based on these radial scans, a B-scan image that captured the path of the CRV through the LC was obtained.

The CRV consists of the central retinal artery and vein, which usually accompany each other; however, there are variations in the way they pass through the LC. In some eyes, there is some distance between the artery and the vein, and assessing them as one vessel bundle is difficult. Therefore, in the present study, we assessed the position of the CRV as the position of the central retinal artery. In eyes where the artery and vein accompanied each other within the LC, the artery and vein were regarded as one vessel bundle (labeled as CRV_VB_ [VB: vessel bundle]), and its position was assessed as well.

### Measurement of parameters

**Figure 2:**
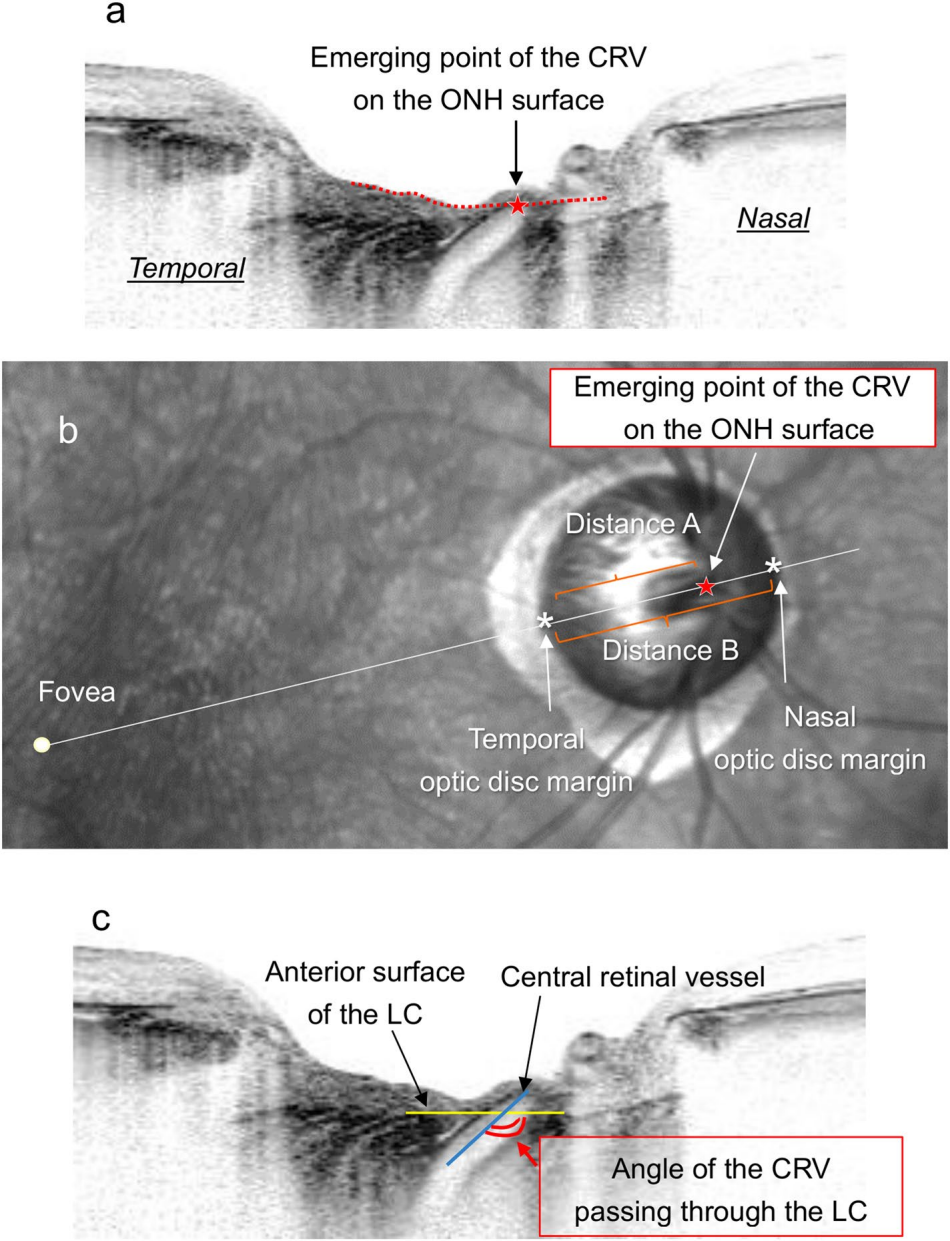
Assessment of the optic nerve head (ONH) parameters. (**a**) Identification of the point of central retinal vessel (CRV) emergence onto the ONH surface by using a B-scan that captured the path of the CRV through the LC. The surface of the ONH is traced by the red dashed line, and the point at which the CRV emerged is presented as a red star. (**b**) Assessment of the position of the CRV on the ONH as the point where the CRV emerged on the ONH surface. The point identified in the B-scan in (**a**) was plotted on the corresponding location on the infrared fundus image (red star). The distance between the temporal optic disc margin and the point of CRV emergence onto the ONH surface was measured on the line connecting the fovea and the emerging point (Distance A). Then, the distance between both sides of the optic disc margin was measured on the same line (Distance B). The position of the CRV on the ONH surface was assessed as a percentage of Distance A to Distance B [(Distance A/ Distance B) × 100]. (**c**) Assessment of the angle of the CRV passing through the LC. It was assessed as an angle between the anterior surface of the LC (yellow line) and the line penetrating the center of the CRV (blue line).

The position of the CRV on the ONH was assessed as the point at which it emerged on the surface of the ONH. This point was identified in the OCT B-scan image that captured the path of the CRV through the LC (Fig. 2a) and was plotted on the corresponding location on the infrared (IR) fundus image (Fig. 2b). The distance between the point of CRV emergence onto the ONH and the temporal optic disc margin was measured on the line connecting the fovea and the emerging point (Distance A in Fig. 2b). Then, the distance between both sides of the optic disc margin was obtained on the same line (Distance B in Fig. 2b). The position of the CRV on the ONH was assessed as a percentage of Distance A to Distance B [(Distance A/ Distance B) × 100]. Therefore, when the point was located more nasally within Distance B, it was presented as a higher percentage. The position of the CRV was assessed after confirming colocalization of the point identified in the OCT images and fundus photographs. This assessment was performed in accordance with the method used in the previous studies^16–18^. In brief, the fundus photograph was imported into the software, and the IR fundus image of the SD-OCT that reduced its transparency was superimposed on the fundus photograph. Then, the optic disc structure identified in the fundus photograph was traced onto the IR image.

The angle of the CRV passing through the LC was measured in the B-scan OCT image that captured its path through the LC (Fig. 2c). This angle was measured as being that between the anterior surface of the LC and the line penetrating the center of the CRV. Therefore, if the CRV took a more nasally angled path through the LC, it was presented to a higher degree.

The depth of the anterior LC surface was measured in the B-scan OCT image obtained in the line connecting the fovea and the point of CRV emergence onto the ONH. This depth was measured using the anterior scleral surface as a reference to eliminate the effect of choroidal thickness^19,20^. The reference line was drawn by connecting two points of the anterior scleral surface located 1,750 μm from the center of the optic disc, because visibility of the scleral surface was reported to be clearest in these locations^21,22^. The anterior LC depth was measured as the length of the perpendicular line from the reference line to the anterior LC surface at its maximally depressed point^23^.

The measurements were performed by 2 investigators (YS and HS) who were masked to the clinical information of the subjects, and cases of disagreement were dealt with by discussion between the 2 investigators to achieve consensus.

### Statistical analyses

Differences in continuous variables between groups were assessed by using the *t*-test or Mann–Whitney *U*-test, depending on the linearity of the Q-Q plots. The relationship between the position of the CRV on the ONH and possible confounders was assessed by using multiple regression analyses. Interobserver reproducibility of the measurement of the ONH parameters was assessed by two independent observers (YS and HS) in a separate group of 30 glaucoma eyes, and the corresponding intraclass correlation coefficients (ICCs) and 95% confidence intervals (CIs) were calculated. Statistical analyses, with two-sided *P-*values, were performed using SPSS software version 25 (SPSS, Chicago, IL, USA). The level of significance was set at *P* < 0.05.

## Results

From 256 eyes of 128 patients with glaucoma, 62 eyes of 47 patients were excluded for the following reasons: poor-quality OCT images (n = 32), unreliable VF test results (n = 16), ocular diseases other than glaucoma that might affect the VF (n = 9), and congenital optic disc anomalies or suspected anomalies (n = 5). One eye was eligible in 32 subjects, and both eyes were eligible in 81 subjects. From the subjects for whom both eyes were eligible, one eye was randomly chosen. As a result, 113 eyes of 113 patients were included in the analysis. Sixty eyes of 60 normal subjects were included as controls. Among the included subjects, 77 glaucomatous eyes and 37 normal eyes were chosen for the CRV_VB_ analysis.

The ICCs (95% CIs) for the interobserver reproducibility of the position of the CRV on the ONH, angle of the CRV passing through the LC, and anterior LC depth were 0.90 (0.87–0.94), 0.91 (0.88–0.95), and 0.91 (0.88–0.94), respectively.

Among the 113 included subjects with glaucoma, 56 (49.6%) were men, and the mean age was 66.3 ± 12.7 years (Table 1). The average mean deviation (MD) of the Humphrey VF test was -12.31 ± 8.81 decibel. There was no statistically significant difference in age, IOP, refractive status, and central corneal thickness between eyes with glaucoma and normal eyes.

**Table 1:** Demographic data of the subjects.

	Glaucoma patients (n = 113)	Normal subjects (n = 60)	*P* Value	*P* Value^†^
Sex (male/female)	56/57	25/35	0.3250*	> 0.05
Age (yrs)	66.3 ± 12.7	65.0 ± 12.1	0.1965*	> 0.05
Intraocular pressure: untreated (mmHg)	25.1 ± 11.4	n/a	n/a	n/a
imaging day (mmHg)	15.3 ± 4.0	14.6 ± 2.9	0.1866**	> 0.05
Spherical equipment (diopter)	− 0.55 ± 1.64	− 0.57 ± 1.30	0.9135**	> 0.05
Axial length (mm)	23.43 ± 1.16	23.38 ± 0.91	0.7673**	> 0.05
Central corneal thickness (μm)	538.4 ± 33.2	548.7 ± 35.1	0.0589*	> 0.05
Retinal nerve fiber layer thickness (μm)	58.9 ± 15.1	99.6 ± 9.4	< 0.0001**	< 0.0001
MD of the Humphrey VF test (decibel)	− 12.31 ± 8.81	− 0.26 ± 1.41	< 0.0001**	< 0.0001
Position of the CRV on the optic nerve head (%)	66.0 ± 8.6	54.3 ± 9.5	< 0.0001**	< 0.0001
Angle of the CRV passing through lamina cribrosa (degree)	137.9 ± 17.2	114.9 ± 18.2	< 0.0001**	< 0.0001
Anterior lamina cribrosa depth (μm)	382.7 ± 126.4	248.9 ± 73.2	< 0.0001**	< 0.0001

Values are shown in means ± standard deviations. *P* values < 0.05 are noted in boldface.

For position of the CRV on the optic nerve head, greater percentage indicates more nasal displacement.

For angle of the CRV passing through lamina cribrosa, greater degree indicates more nasally angled path.

* Student *t* test. **Mann–Whitney *U* test. ^†^ *P* value after Bonferroni correction.

n/a, not applicable; MD, mean deviation; VF, visual field; CRV, central retinal vessel.

**Figure 3:**
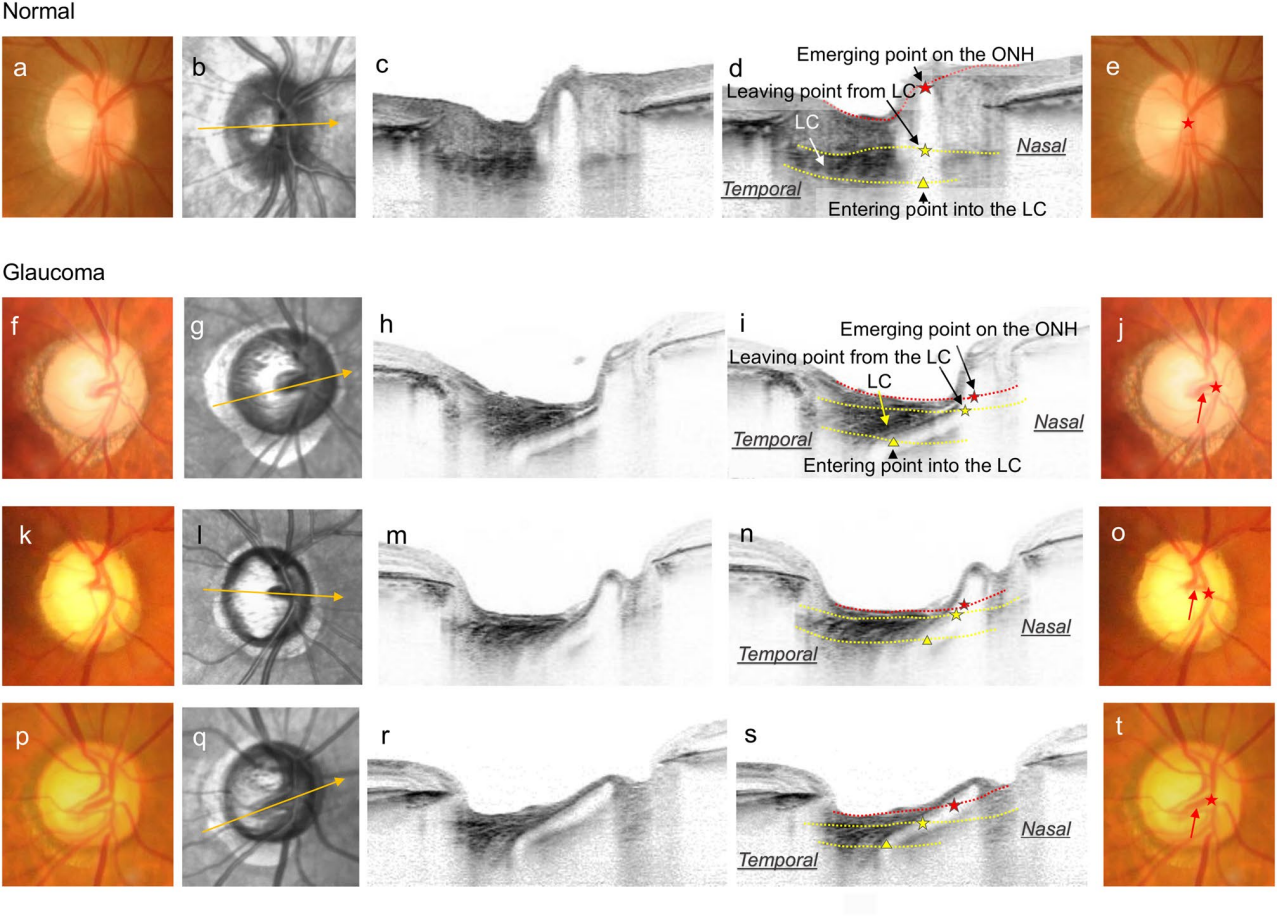
Spectral-domain optical coherence tomography (SD-OCT) of the optic nerve head (ONH) showing the path of the central retinal vessel (CRV) through the lamina cribrosa (LC). (**a–e**) Normal eye. (**a**) Fundus photograph. (**b**) Infrared fundus image with the SD-OCT scan line (orange arrow). (**c**) SD-OCT B-scan image obtained at the line shown in (**c**). (**d**) The same image as (**c**) but with labels. The red dashed line shows the surface of the ONH and the red star shows the point where the CRV emerges on it. The yellow star indicates the point where the CRV leaves the LC, and the yellow triangle indicates the point where it enters the LC from retrolaminar ONH region. In normal eyes, the CRV takes an almost perpendicular path through the LC. (**e**) Same image as (**a**) but with labels. The position of the CRV on the ONH (red star) was identical to the point of CRV emergence onto the ONH, identified in **d** (red star). (**F–t**) Glaucomatous eyes. (**f, k, p**) Fundus photographs. (**g, l, q**) Infrared fundus images of **f**, **k**, **p**, respectively, with the OCT scan lines (orange arrows). (**h, m, r**) SD-OCT images of the ONH that captured the path of the CRV through the LC obtained at the scan lines shown in (**g**, **l**, **q**), respectively. (**i, n, s**) The same images as (**h**, **m**, and **r**), respectively, but with the same labels as those used in (**d**). In eyes with glaucoma, the CRV takes an angled path through the LC toward the nasal direction and emerges on the ONH surface at its nasal area. The CRV and surrounding laminar pores showed similar angles and exhibited parallel alignment as if they deformed simultaneously. (**j, o, t)** Same images as (**f**, **k**, and **p**), respectively, but with labels. The nasal position of the CRV on the ONH (red star) is identical to the point of CRV emergence onto the ONH surface identified in the OCT B-scan. The nasally angled path of the CRV within the LC is observed in the fundus photograph as a traversing vessel on the ONH (red arrow).

In normal eyes, the CRV usually took an almost perpendicular path through the LC (Fig. 3a–e). However, in eyes with glaucoma, the CRV took an angled path toward the nasal direction (Fig. 3f–t). The CRV entered the LC from the retro-laminar ONH region around its central area, changed its direction from perpendicular to nasal, and left the LC to the prelaminar ONH region at its nasal area. The CRV and surrounding laminar pores presented similar angles, and they exhibited parallel alignment as if they deformed simultaneously. The nasally angled path of the CRV through the LC was observed in the fundus photograph as a traversing vessel on the ONH visible through the translucent overlying tissue (red arrows in Fig. 3j,o,t).

The position of the CRV on the ONH was significantly displaced nasally in eyes with glaucoma compared with normal eyes (66.0 ± 8.6% vs. 54.3 ± 9.5%, *P* < 0.0001, Table 1). The CRV passing through the LC was significantly more nasally angled in eyes with glaucoma (137.9 ± 17.2 degrees vs. 114.9 ± 18.2 degrees, *P* < 0.0001). The anterior LC depth was significantly deeper in eyes with glaucoma than in normal eyes (382.7 ± 126.4 μm vs. 248.9 ± 73.2 μm, *P* < 0.0001).

**Figure 4:**
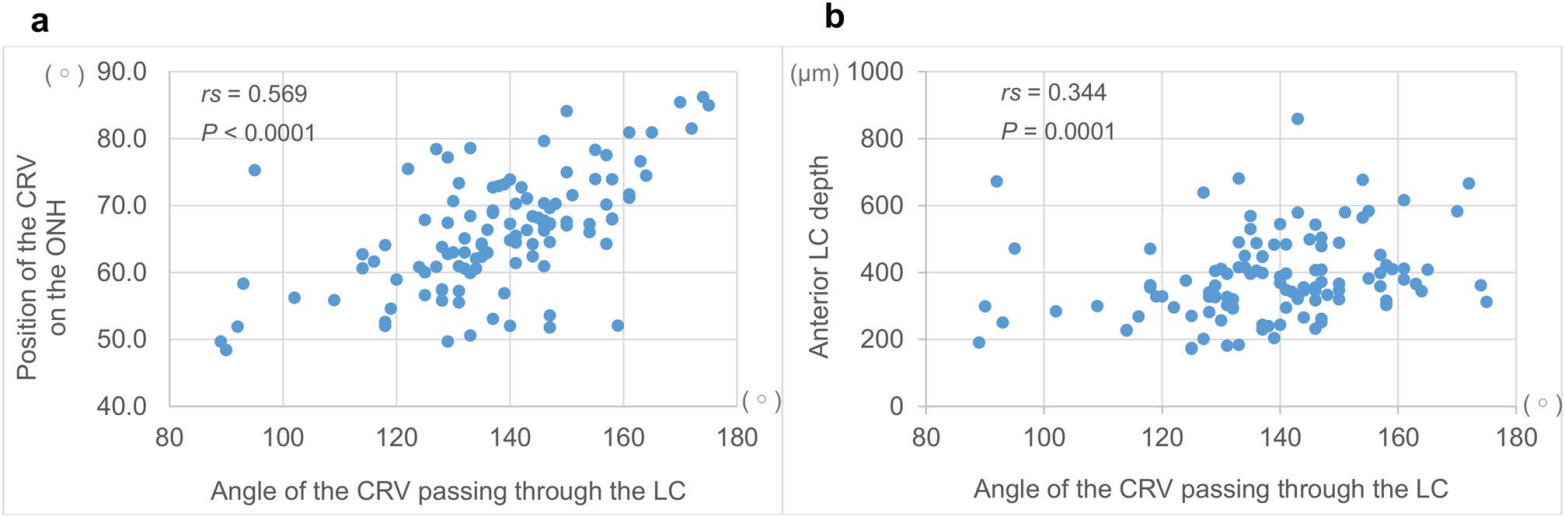
(**a**) Correlation between the angle of the central retinal vessel (CRV) passing through the lamina cribrosa (LC) and its position on the optic nerve head (ONH) in eyes with glaucoma. (**b**) Correlation between the angle of the CRV passing through the LC and the anterior LC depth in eyes with glaucoma. A higher degree of the angle of the CRV passing through the LC indicates a more nasally angled path, and higher percentage of the position of the CRV on the ONH indicates a more nasal position of it.

In eyes with glaucoma, the position of the CRV on the ONH was significantly correlated with the angle of the CRV passing through the LC (*rs* = 0.569, *P* < 0.0001, Fig. 4a), which indicated that eyes with a more nasally angled CRV through the LC exhibited more nasal displacement of the CRV on the ONH. In addition, the anterior LC depth was significantly correlated with the angle of the CRV passing through the LC (*rs* = 0.344, *P* = 0.0001, Fig. 4b), which indicated that eyes with a more nasally angled CRV through the LC exhibited a deeper anterior LC depth.

In eyes with glaucoma, the position of the CRV on the ONH was significantly negatively correlated with the MD of the Humphrey VF test and age after considering the effect of possible confounders (Table 2). This indicated that eyes with worse glaucomatous VF defects and eyes of younger patients exhibited significantly more nasal displacement of the CRV on the ONH. The position of the CRV also exhibited significant correlation with the retinal nerve fiber layer thickness when it was included in the analysis instead of the MD (Supplementary Table S1).

**Table 2:** Relationship between the position of the central retinal vessel on the ONH and possible confounders: Results of multiple regression analysis.

	Univariate analysis Regression coefficient (95% confidence intervals)	Multivariate analysis Standard regression coefficient (95% confidence intervals)
Mean deviation	− 0.270 (− 0.449 to − 0.087)	**− 0.328 (− 0.493 to − 0.148)**
Age	− 0.367 (− 0.542 to − 0.193)	**− 0.329 (− 0.373 to − 0.103)**
Axial length	0.300 (0.120 to 0.479)	0.156 (− 0.318 to 3.025)
IOP: untreated	− 0.078 (− 0.109 to 0.266)	− 0.101 (− 0.211 to 0.060)
: on imaging day	0.090 (− 0.097 to 0.278)	0.074 (− 0.204 to 0.5241)
Sex	− 0.208 (− 0.392 to − 0.024)	− 0.076 (− 4.377 to 1.799)

In the multiple regression analysis, all variables indicated are used as independent variables.

Significant coefficients are noted in boldface.

ONH, optic nerve head; IOP, intraocular pressure.

*R*^*2*^*,* contribution ratio was 0.270.

In the analysis of the CRV_VB_, the position of the CRV_VB_ on the ONH was significantly displaced nasally in eyes with glaucoma than in normal eyes (63.5 ± 7.7% vs. 53.8 ± 6.4%, *P* < 0.0001, Supplementary Table S2). In the multiple regression analysis, this position was significantly negatively correlated with the MD (Supplementary Table S3).

## Discussion

In the present study, we reported significant nasal displacement of the CRV on the ONH in eyes with glaucoma compared with normal eyes. More nasal displacement of the CRV on the ONH was associated with its more nasally angled path through the LC. The extent of vessel displacement was significantly associated with the severity of the glaucomatous VF defect. Assessment using the CRV_VB_ exhibited the same results. To the best of our knowledge, this is the first study to demonstrate nasal displacement of the CRV on the ONH in glaucoma in association with its nasally angled path through the LC by using SD-OCT.

The present results confirm that nasal displacement of the CRV on the ONH is associated with glaucoma. This is because the nasal displacement was significantly greater in eyes with glaucoma than in normal eyes, and its extent was significantly associated with the severity of the glaucomatous VF defect. A previous study examined nasal displacement of the CRV on the ONH by using fundus photographs^4^, and reported that the extent of displacement was not significantly different between glaucomatous and normal eyes. That study defined nasal displacement of the CRV as the distance between the point of CRV emergence onto the cup floor and the major vessel trunk on the ONH. Therefore, the prelaminar part of the CRV was thought to be assessed. At the emerging point on the cup floor, the CRV was already displaced nasally after taking an angled path through the LC. It might not be displaced very much after that on the ONH, and the extent of displacement might be similar to that in normal eyes. On the other hand, the present study used SD-OCT and defined nasal displacement of the CRV as a percentage of the distance from the temporal disc margin to the point of CRV emergence onto the ONH. With this method, we were able to demonstrate significant nasal displacement of the CRV in eyes with glaucoma compared with normal eyes. The result of the multiple regression analysis also showed that the position of the CRV was negatively associated with age, which implied that the extent of nasal displacement of the CRV was significantly smaller in the eyes of older patients. This finding is in accordance with the results of previous studies that demonstrated a smaller ONH deformation in the eyes of older patients^24–26^. It is suggested that the smaller ONH deformation is caused by the stiffer connective tissue in aged eyes^26^. This might explain the present result which showed less nasal displacement of the CRV on the ONH in the eyes of older patients.

The results of the present study suggested that the nasally angled path of the CRV within the LC occurred along with the glaucomatous deformation of the LC. This was because the CRV within the LC exhibited a parallel arrangement to the surrounding laminar pores, which indicated that they were depressed and angled simultaneously under the IOP-related stress (Fig. 3). The nasally angled path of the CRV within the LC was identified in the fundus examination as a traversing vessel on the ONH. LC deformation is difficult to see in many eyes, whereas traversing vessels on the ONH are easy to identify. This may serve as a clinical surrogate to detect glaucomatous LC deformation. The LC is considered to be a primary site of glaucomatous axonal injury^27–29^, and characteristic deformations of the LC, such as posterior displacement and thinning, have been reported^13,15,22,23,30^. In the present study, eyes with a more angled CRV within the LC exhibited significantly greater posterior LC displacement, that is, a greater anterior LC depth. These findings suggested that nasal angling of the CRV through the LC occurred concomitantly with the posterior displacement of the LC as part of the whole glaucomatous LC deformation.

**Figure 5:**
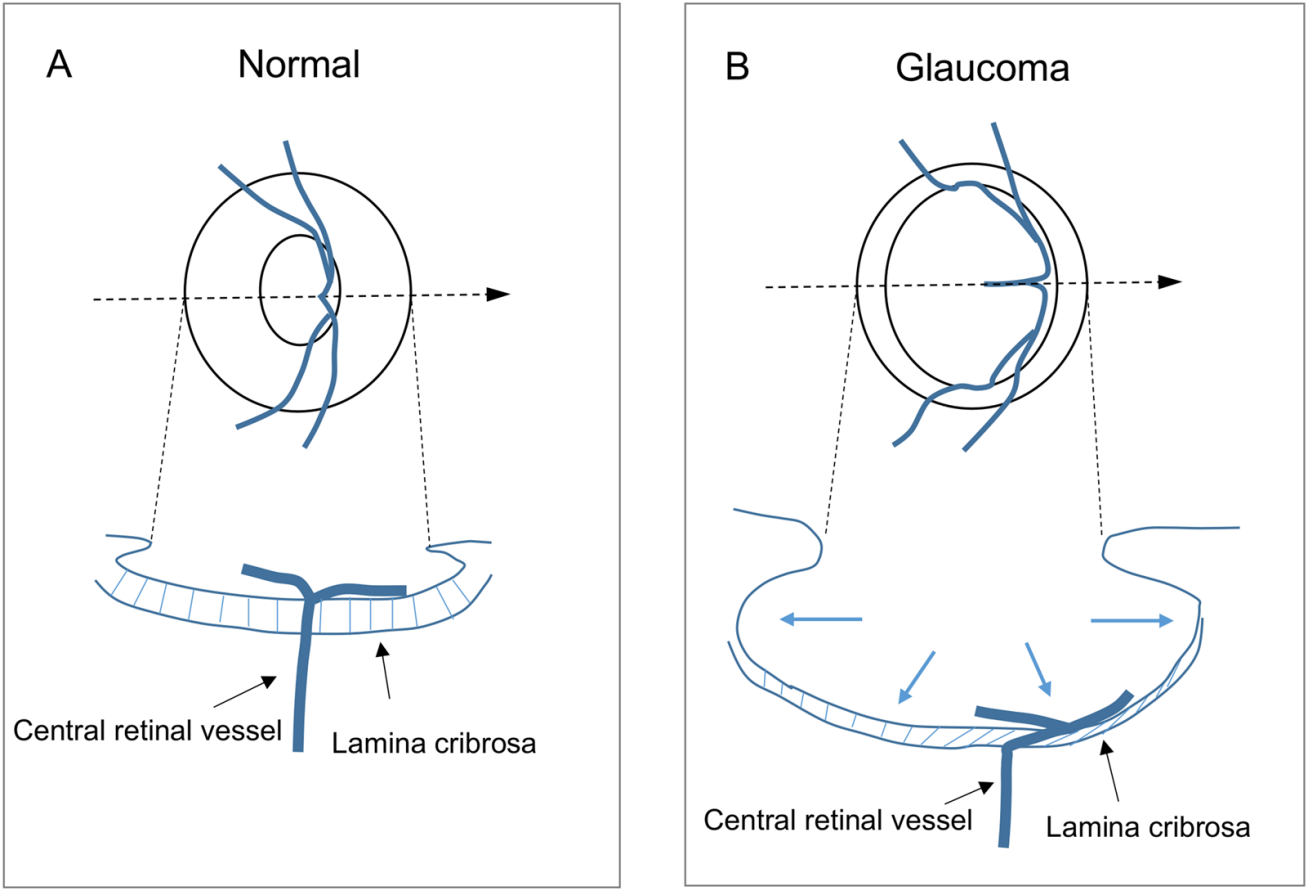
Scheme of the mechanism behind the nasal displacement of the central retinal vessel on the optic nerve head in eyes with glaucoma.

The possible mechanism behind the nasal displacement of the CRV on the ONH in eyes with glaucoma may be explained as follows (Fig. 5). In normal eyes, the CRV enters the LC from the retrolaminar ONH region at its central area and takes a relatively perpendicular path through the LC. When glaucoma develops in an eye, IOP-related stress is placed on the ONH, and the LC is displaced posteriorly and becomes thinner. The laminar pores are depressed and become angled during these deformations. The material of the retinal vessel is supposed to be softer than that of the LC tissue; therefore, when the surrounding LC tissue is angled, the CRV embedded within the LC is angled simultaneously. A greater angle of the CRV causes a longer nasal transverse distance through the LC and induces greater nasal displacement of the CRV on the ONH.

The present study has several limitations. It included mostly emmetropic eyes to eliminate the effect of myopic ONH deformation from the results^9–11,30^. Whether nasal displacement of the CRV on the ONH is also evident in myopic eyes requires further study. In addition, this study included only Japanese subjects; therefore, whether the present findings are applicable to other ethnicities needs to be elucidated.

In conclusion, we herein provide new evidence to confirm that nasal displacement of the CRV on the ONH is associated with glaucoma by using SD-OCT. The nasal displacement of the CRV is induced by a nasally angled passage through the LC, which is considered to occur along with glaucomatous LC deformation.

## Supplementary Information

Supplementary File 1.

Supplementary File 2.

Supplementary File 3.
