# Supplementary material for: Nasal displacement of retinal vessels on the optic disc in glaucoma associated with a nasally angled passage through lamina cribrosa
Source: Sci Rep. 2021 Feb 18;11:4176. doi: 10.1038/s41598-021-83720-0 (PMC7892818; doi:10.1038/s41598-021-83720-0)
Supplement: Supplementary file 1 [file 41598_2021_83720_MOESM1_ESM.pdf]

Table S1. Relationship between the position of the central retinal vessel on the ONH and possible confounders: Results of multiple regression analysis

	Univariate analysis	Multivariate analysis
	Regression coefficient (95% confidence intervals)	Standard regression coefficient (95% confidence intervals)
**RNFL thickness**	**-0.285 (-0.465 ~ -0.104)**	**-0.266 (-0.438 ~ -0.094)**
**Age**	**-0.367 (-0.542 ~ -0.193)**	**-0.273 (-0.462 ~ -0.084)**
Axial length	**0.300 (0.120 ~ 0.479)**	0.160 (-0.036 ~ 0.356)
IOP: untreated	-0.078 (-0.109 ~ 0.266)	-0.035 (-0.210 ~ 0.140)
: on imaging day	0.090 (-0.097 ~ 0.278)	0.066 (-0.105 ~ 0.237)
Sex	-0.208 (-0.392 ~ -0.024)	-0.080 (-0263 ~ 0.013)

In the multiple regression analysis, all variables indicated are used as independent variables.
Significant coefficients are noted in boldface.
ONH, optic nerve head; RNFL, retinal nerve fiber layer; IOP, intraocular pressure.
R^2, contribution ratio was 0.244.
