# Supplementary material for: Nasal displacement of retinal vessels on the optic disc in glaucoma associated with a nasally angled passage through lamina cribrosa
Source: Sci Rep. 2021 Feb 18;11:4176. doi: 10.1038/s41598-021-83720-0 (PMC7892818; doi:10.1038/s41598-021-83720-0)
Supplement: Supplementary file 2 [file 41598_2021_83720_MOESM2_ESM.pdf]

Table S2. Demographic data of the subjects

	Glaucoma patients (n = 77)	Normal subjects (n = 37)	*P* Value	*P* Value†
Sex (male/female)	40/37	19/18	0.9529*	> 0.05
Age (yrs)	66.9 ± 12.2	65.1 ± 11.5	0.4335*	> 0.05
Intraocular pressure: untreated (mmHg)	24.9 ± 11.5	n/a	n/a	n/a
imaging day (mmHg)	15.1 ± 4.4	14.9 ± 3.1	0.7402**	> 0.05
Spherical equipment (diopter)	-0.42 ± 1.64	-0.43 ± 1.30	0.9453**	> 0.05
Axial length (mm)	23.45 ± 0.98	23.41 ± 0.69	0.8022**	> 0.05
Central corneal thickness (µm)	537.3 ± 31.0	554.6 ± 37.8	0.0106*	> 0.05
**Retinal nerve fiber layer thickness (µm)**	**59.4 ± 14.5**	**97.6 ± 6.8**	**< 0.0001****	**< 0.0001**
**MD of the Humphrey VF test (decibel)**	**-11.63 ± 8.76**	**-0.29 ± 1.62**	**< 0.0001****	**< 0.0001**
**Position of the CRV_{VB} on the optic nerve head (%)**	**63.5 ± 7.7**	**53.8 ± 6.4**	**< 0.0001****	**< 0.0001**
**Angle of the CRV_{VB} passing through lamina cribrosa (degree)**	**136.8 ± 15.8**	**114.1 ± 19.0**	**< 0.0001****	**< 0.0001**
**Anterior lamina cribrosa depth (µm)**	**383.2 ± 123.5**	**252.6 ± 70.6**	**< 0.0001****	**< 0.0001**

Values are shown in means ± standard deviations. *P* values < 0.05 are noted in boldface.

For position of the CRV_{VB} on the optic nerve head, greater percentage indicates more nasal displacement.

For angle of the CRV_{VB} passing through lamina cribrosa, greater degree indicates more nasally angled path.

* Student *t* test. **Mann-Whitney *U* test. † *P* value after Bonferroni correction.

n/a, not applicable; MD, mean deviation; VF, visual field; CRV_{VB}, central retinal vessel assessed as a vessel bundle.
