# Supplementary material for: Nasal displacement of retinal vessels on the optic disc in glaucoma associated with a nasally angled passage through lamina cribrosa
Source: Sci Rep. 2021 Feb 18;11:4176. doi: 10.1038/s41598-021-83720-0 (PMC7892818; doi:10.1038/s41598-021-83720-0)
Supplement: Supplementary file 3 [file 41598_2021_83720_MOESM3_ESM.pdf]

Table S3. Relationship between the position of the CRV_{VB} on the ONH and possible confounders: Results of multiple regression analysis

	Univariate analysis	Multivariate analysis
	Regression coefficient (95% confidence intervals)	Standard regression coefficient (95% confidence intervals)
**Mean deviation**	**-0.420 (-0.629 ~ -0.211)**	**-0.433 (-0.644 ~ -0.221)**
**Age**	**-0.306 (-0.525 ~ -0.087)**	**-0.289 (-0.499 ~ -0.079)**
Axial length	0.161 (-0.066 ~ 0.388)	0.091 (-0.118 ~ 0.310)
IOP: untreated	**0.291 (0.071 ~ 0.511)**	0.091 (-0.121 ~ 0.303)
: on imaging day	0.132 (-0.097 ~ 0.360)	0.122(-0.078 ~ 0.323)
Sex	-0.166 (-0.393 ~ -0.061)	-0.035 (-0.247 ~ 0.177)

In the multiple regression analysis, all variables indicated are used as independent variables.
Significant coefficients are noted in boldface.
CRV_{VB}, central retinal vessel assessed as a vessel bundle; ONH, optic nerve head;
IOP, intraocular pressure.
R^2, contribution ratio was 0.334.
